# Tau oligomers impair memory and induce synaptic and mitochondrial dysfunction in wild-type mice

**DOI:** 10.1186/1750-1326-6-39

**Published:** 2011-06-06

**Authors:** Cristian A Lasagna-Reeves, Diana L Castillo-Carranza, Urmi Sengupta, Audra L Clos, George R Jackson, Rakez Kayed

**Affiliations:** 1 George P. and Cynthia Woods Mitchell Center for Neurodegenerative Diseases, Departments of Neurology, and Neuroscience and Cell Biology, The University of Texas Medical Branch, Galveston, TX 77555-1045, USA

## Abstract

**Background:**

The correlation between neurofibrillary tangles of tau and disease progression in the brains of Alzheimer's disease (AD) patients remains an area of contention. Innovative data are emerging from biochemical, cell-based and transgenic mouse studies that suggest that tau oligomers, a pre-filament form of tau, may be the most toxic and pathologically significant tau aggregate.

**Results:**

Here we report that oligomers of recombinant full-length human tau protein are neurotoxic in vivo after subcortical stereotaxic injection into mice. Tau oligomers impaired memory consolidation, whereas tau fibrils and monomers did not. Additionally, tau oligomers induced synaptic dysfunction by reducing the levels of synaptic vesicle-associated proteins synaptophysin and septin-11. Tau oligomers produced mitochondrial dysfunction by decreasing the levels of NADH-ubiquinone oxidoreductase (electron transport chain complex I), and activated caspase-9, which is related to the apoptotic mitochondrial pathway.

**Conclusions:**

This study identifies tau oligomers as an acutely toxic tau species in vivo, and suggests that tau oligomers induce neurodegeneration by affecting mitochondrial and synaptic function, both of which are early hallmarks in AD and other tauopathies. These results open new avenues for neuroprotective intervention strategies of tauopathies by targeting tau oligomers.

## Introduction

The major biological functions of the microtubule-associated protein tau, include: microtubule assembly, axonal transport, neurite outgrowth, and stability of microtubules [1]. Most of the biological functions of tau are modulated by site-specific phosphorylation [2]. Tau self-assembly, aggregation, and accumulation in neurofibrillary tangles (NFTs) are hallmarks of Alzheimer's disease (AD) and other neurodegenerative diseases [3,4]. Although the importance of tau in AD and other tauopathies is well-established [5-7], unanswered is whether NFTs are the primary neurotoxic factor. Most research has focused on NFTs because of the reported correlation between NFTs and disease progression in the brains of AD patients [8-10]. However, recent data suggest that soluble pre-filament forms of tau may be the most toxic and pathologically significant forms of tau aggregates [11,12]. Cell death and synaptic lesions occur independently of formation of NFTs in h-tau mice expressing non-mutant human tau [13,14]. Hippocampal synapse loss and microgliosis precede formation of NFTs in the P301S transgenic mouse model (P301S Tg) [15]. Moreover, tau oligomers were biochemically characterized in a conditional model (rTg4510) expressing the P301L h-tau mutant. Surprisingly, the best correlate of neuronal loss and behavioral deficits in these models was the accumulation of oligomeric tau, whereas there was no relation with NFTs [16,17]. In addition, stereologic studies of human AD show that neuronal loss actually precedes NFT formation [18,19]. Granular tau oligomers were detected and biochemically isolated at very early stages of the disease, when clinical symptoms of AD and NFTs are believed to be absent [20,21], and tau-positive fine granules were found in postmortem tissue from the parkinsonism-dementia complex of Guam (PDC) tauopathy [22]. Even more recently, tau oligomers were detected in platelets from AD patients, suggesting that this species of tau protein could serve as a new biological marker for AD [23].

Mechanistic studies of aggregation of full-length tau protein in vitro revealed that tau aggregates by means of either a nucleation-dependent mechanism [24] or by the formation of intermediates [25]. In the present study, we investigated the neurotoxicity of different forms of tau in vivo by injecting well-characterized oligomers, fibrils, or monomers of full-length recombinant h-tau-441 (2N4R) into the hippocampus of C57BL/6 wild-mice. We found that the mice injected with tau oligomers presented with memory deficits in their novel-object recognition task, which is widely used for evaluating memory in AD mouse models [26-29].

We also determined the loss of synaptic-related proteins and mitochondrial respiratory chain components in conjunction with the activation of the mitochondrial dysfunction markers and the pro-apoptotic protein caspase-9. Our results strongly suggest that accumulation of tau oligomers result in learning impairment through the disruption of synaptic and mitochondrial functions.

## Methods

### Preparation of tau oligomers and fibrils

Recombinant tau protein (tau-441 (2N4R) M.Wt 45.9 kDa) was expressed and purified as described [30,83]. In brief, we transformed the BL21 (DE3) strain of *Escherichia coli *with pET-28 plasmids and grown in LB medium at 37°C under vigorous shaking. At an OD_600 _of 0.4-0.6, we induced protein expression with 1 mM isopropyl β-D-thiogalactoside. Bacteria were incubated for an additional 3 h and then centrifuged for 20 min at 3,500 × *g*. Pellets were resuspended in 500 mM NaCl, 20 mM PIPES (pH 6.5), 1 mM EDTA, and 50 mM 2-mercaptoethanol. After 10 min on ice, we sonicated the samples 3 × 30 sec at power setting seven. We pelleted the bacterial debris for 20 min at 10,000 × *g *and soluble tau protein was precipitated from supernatant by using ammonium sulfate (60%, m/V). After 1 h on ice, samples were centrifuged for 10 min at 10,000 × *g*. Pellets were resuspended in purified H_2_O, 2 mM dithiothreitol (DTT), and loaded onto cation exchange column (Bio-Rad). Protein was eluted from the column in a 0.05-1 M NaCl gradient containing 20 mM PIPES (pH 6.5), 0.5 mM EDTA, and 2 mM DTT. We analyzed the fractions using SDS-PAGE; and we pooled tau protein samples and added 5 mM DTT. The protein was purified using Superdex column (Amersham Pharmacia). Elution occurred with 100 mM NaCl, 10 mM PIPES (pH 6.5), 1 mM EDTA, and 2 mM DTT. Pooled samples of monomeric tau and added to 5 mM DTT. At that point, the protein was >95% pure, as assessed by SDS-PAGE. For storage, tau was precipitated overnight on ice with an equal volume of MetOH, centrifuged for 20 min at 10,000 × *g*, washed once with MetOH and 2 mM DTT, and stored at -80°C. Protein concentrations were determined at 276 nm in 6 M guanidinium chloride. We treated the recombinant tau with 8 M urea to obtain monomeric tau, then dialyzed overnight against 1 × phosphate-buffered saline (PBS), pH 7.4, normalized to 1 mg/mL with PBS and aliquots of tau monomer in PBS were kept at -20°C.

Tau oligomers were prepared as previously described [36]. Specifically, 300 μL of the tau stock (1 mg/mL) was added to 700 μL of PBS 1 ×, final concentration (0.3 mg/mL). Seven microliters of β42 amyloid (Aβ42) oligomers (0.3 mg/mL) was added as seeds and the sample was mixed by pipetting for 1 min. We incubated the sample at room temperature for 1 h on an orbital shaker. We purified the resulting tau oligomers by fast protein liquid chromatography (FPLC) and used them to seed a fresh sample of monomeric tau; after two rounds of seeding monomeric tau with purified tau oligomers, all Aβ oligomer seeds are eliminated. The samples have no Aβ as tested by either enzyme-linked immunosorbent assay (ELISA) or Western blotting using 4G8. This is not surprising since we used (1:140) Aβ/tau ratio in the first round, which is less than the standard used in the literature as seeds to promote aggregation. After three rounds, there was less than 1:2,470,000 Aβ/tau, which is below the detection limits of the methods available. To prepare the fibrils, tau oligomers were allowed to mix 1-2 days on the orbital shaker. Tau preparations were characterized by atomic force microscopy (AFM) by a non-contact tapping method (ScanAsyst-air) using a Multimode 8 AFM machine (Veeco, CA), and purified by size-exclusion chromatography, using an LC-6AD Shimadsu high-performance liquid chromatography (HPLC) system fitted with a TSK-GEL G3000 SWXL (30 cm × 7.8 mm) column, Supelco-808541. PBS, pH 7.4, was used as the mobile phase, flow rate 0.5 mL/min. Gel filtration standard (Bio-Rad 51-1901) was used for calibrations.

### Animals

Thirty-six male C57BL/6 mice (body weight: 30 ± 2 g) were used at the age of 20 weeks. The mice had free access to food and water and were maintained on a 12-h dark-light cycle in a controlled temperature room (25°C ± 2°C) for the duration of the study. The animals were divided into three groups of twelve mice. In one group, mice were injected with tau oligomers and PBS; in a second group, mice were injected with tau fibrils and PBS; in the third group, mice were injected with tau monomers and PBS. All animal experiments were performed in accordance with IACUC approved protocols.

### Tau subcortical stereotaxic injection

We anesthetized male C57BL/6 mice with ketamine (10 mg/mL) and xylazine (1.5 mg/mL), and then positioned on a stereotaxic frame. Once we identified bregma and drilled holes in that location, 1 μL of 0.9 mg/mL of various tau preparations and/or the same volume of PBS buffer were injected into the hippocampus in the left and right hemispheres, respectively (-2.06 mm posterior, +/-1.75 mm lateral, and 2.5 mm ventral to the bregma) at a rate of 0.2 μL/min.

### Object recognition task

Mice were tested in an open-square white arena, 60 × 60 cm, 40 cm high. The following objects were used: a black metal cylinder, 6 × 7 cm; an orange disk, 1.5 × 5 cm; and a plastic cube, 4 × 4 cm. The task started with a habituation trial, during which the animals were placed in the empty arena for 10 min. The next day, mice were injected with the corresponding tau species and/or the vehicle (PBS). Twenty-four hours later, the mice were again placed in the same arena containing two identical objects (familiarization phase). Exploration was recorded in a 10-min trial. Sniffing, touching, and stretching the head toward the object at a distance of no more than 2 cm were scored as object investigation [29]. Six hours later (test phase), mice were again placed in the arena containing two objects: one identical to one of the objects presented during the familiarization phase (familiar object), and a new, different object (novel object). The time spent exploring the two objects was recorded for 10 min. Memory was expressed as a discrimination index, namely (seconds on novel-seconds on familiar)/(seconds on novel + seconds on familiar) and was expressed as the percentage of time on each object. Animals with no memory impairment spend a longer time investigating the novel object compared with the familiar object, giving a higher discrimination index. At the end of the experiment, we euthanized the mice using cervical dislocation and dissected the brains of six animals per group. We then fixed these brains in 4% buffered paraformaldehyde, and embedded them in paraffin for histological analysis. The brains from the other six mice of each group were frozen for biochemical analysis.

### Western blot analysis

We divided each brain into hemispheres and then the hippocampus was isolated and homogenized in ice-cold PBS with protease inhibitors (5% w/v). We centrifuged the homogenates for 10 min at 300 *g*. Next, we quantified the proteins in the supernatant using a Bradford assay (Sigma, B6916) and separated equal amounts of them using an SDS-PAGE and then we transferred them onto nitrocellulose. After being blocked with non-fat dried milk, membranes were probed with anti-synaptophysin (1:1000, Abcam, ab8049), anti-complex V d-subunit (1:2000, Invitrogen, 459000), anti-complex I subunit (1:1000, Invitrogen 459210), anti-caspase-9 (1:4000, Abcam, ab8087), anti-tubulin (1:1500, Oncogene, CP06), anti-synapsin-1 (1:3000, Abcam, ab8), anti-septin 11 (1:2000, Abcam, ab86268), anti-active caspase-3 (1:1000, Abcam, ab2302), and anti-caspase-8 antibody (1:4000, Abcam, ab52183). The first five antibodies immunoreactivity was detected with horseradish peroxidase (HRP)-conjugated anti-mouse IgG (1:3000, Jackson ImmunoResearch Laboratories, Inc. West Grove, PA) and anti-rabbit IgG (1:3000, Jackson ImmunoResearch Laboratories, Inc., West Grove, PA) for other the four antibodies, followed by electrochemiluminescence (Thermo Scientific, Rockford, IL).

### Immunohistochemical and microscopic analysis

Paraffin sections were deparaffinized, rehydrated, and washed in 0.01 mol/L PBS for 5 min., repeated three times. After blocking in normal goat serum for 1 h, sections were incubated overnight with mouse monoclonal anti-synaptophysin antibody (predilute; Chemicon, IHCR1011-6) or mouse monoclonal (2-23) antibody to caspase-9 (1:200; Abcam, ab78087) or mouse monoclonal anti-NeuN antibody (1:1000, Chemicon, MAB377). The next day the sections were washed in PBS three times for 10 min and then incubated with goat anti-mouse IgG alexa488 (1:700; Invitrogen, A11004) for 1 h. Later, we washed the sections three times for 10 min in PBS, and finally we applied VECTASHIELD mounting medium with DAPI staining (Vector Lab., H-1000) and the slide was coverslipped and sealed with clear nail polish. Double staining was performed, using the mouse monoclonal antibody HT7 (1:1000; Pierce, MN1000), which recognizes human tau (but not mouse tau) [50] and the rabbit polyclonal antibody anti-porin (1:500; Abcam, ab15895), a marker for mitochondria. The secondary antibodies goat anti-mouse IgG alexa488 (1:700; Invitrogen, A11004) and goat anti-rabbit IgG alexa568 (Invitrogen, A11036) were used to detect HT7 and anti-porin, respectively. In order to determine degenerating neurons, we stained the sections with Fluoro-Jade B according to manufacturer specifications (Millipore, AG310). Next, we examined the brain sections using an epifluorescence microscope (Nikon Eclipse 800) equipped with a CoolSnap-FX monochrome CCD camera (Photometrics, Tucson, AZ) using a standard Nikon FITC and DAPI filters and acquired and analyzed the images using the Metavue V7.1 software (Molecular Devices, Downingtown, PA). To examine nuclear morphology and degeneration, we performed hematoxylin staining and acquired bright field images using a Nikon Multizoom AZ100 microscope equipped with a Nikon DS-2M color CCD camera (Nikon Instruments Inc, Melville, NY).

### Intensity correlation analysis

Intensity correlation analysis (ICA) is based on the principle that for any set of values the sum of the differences from the mean equals zero, i.e., Σ_N _(*Ai *_ *a*) = 0, where *a *is the mean of the distribution with *N *values of *Ai*. In our case, *N *is the number of pixels, and *Ai *is the staining intensity for each pixel. It follows that for *N *pixels associated with two sets of random staining intensities (*Ai *and *Bi*), the sum of the product of their differences will also tend to zero; thus, Σ_N_(*Ai *_ *a*)(*Bi *_ *b*) ≈ 0. However, this is not the case if the two intensities are dependent (when the product tends to be a positive value) or if they are segregated (when the product tends to be a negative value). Thus, with dependent staining Σ_N _(*A i-a*)(*Bi*-*b*) > 0, whereas with segregated staining Σ_N_(*Ai*-*a*)(*Bi*-*b*) < 0 [51]. We used this property to test for dependent or segregated staining between HT7 and anti-porin antibody. Analysis performed using Image-J software (National Institutes of Health).

### Statistical analysis

For protein quantification, the densitometry of each band in the Western blot was normalized with tubulin. All densitometry results represent the mean and standard deviations of all of the determinations performed. Data were compared by one-way analysis of variance (ANOVA) followed by Bonferroni's multiple comparison test. The criterion for statistical significance between groups was P < 0.001. The same analysis was performed for the quantification of Fluoro-Jade B positive cells. In the case of the object recognition experiments, the discrimination index data was analyzed using one-way ANOVA, followed by a Tukey's post-hoc test. The criterion for statistical significance was p < 0.01. We analyzed the percent of time spent with each object using Student's t-test. Specifically the time spent with the novel object was compared with the time spent with the familiar object in the same group. The criterion for statistical significance was p < 0.001. All statistical analyses were performing using OriginPro 8.0 software.

## Results

### Tau oligomers impair memory encoding/consolidation

**Figure 1:**
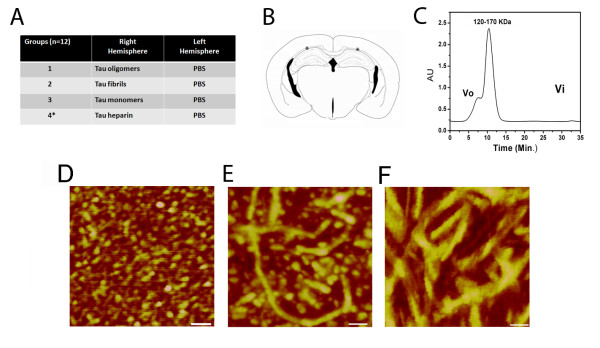
**Experimental design and characterization of tau samples**. (A) Mice were divided into four groups, one injected with tau oligomers and PBS, a second group injected with tau fibrils and PBS a third group injected with tau monomers and PBS, and a fourth group injected with tau heparin fibrils and PBS used as a control (*). (B) Schematic representation of a coronal section of a mouse brain with * indicating the injection sites of the tau preparations in PBS. (C) FPLC profile of tau oligomers injected into mice brains. (D) AFM image showing homogeneous population of tau oligomers (scale bar = 140 nm). (E) AFM image of tau fibrils (scale bar = 70 nm). (F) AFM images of tau fibrils prepared with heparin (scale bar = 50 nm).

**Figure 2:**
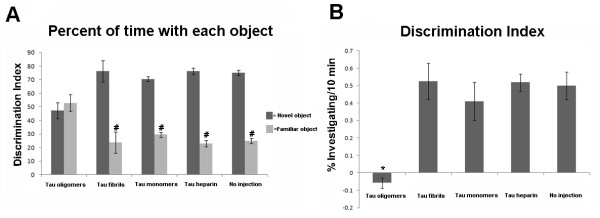
**Tau oligomers impaired the recognition memory in mice**. (A) Effects of tau oligomers, fibrils, and monomers on memory were investigated in C57BL/6 mice by using the object recognition task. Histograms indicate the percentage of exploration of the familiar and novel objects. Mice injected with monomers or fibrils spent significantly more time investigating the novel object versus mice injected with tau oligomers, indicating impaired memory, as shown by their inability to recognize the familiar object. #p < 0.01 vs. novel object. (B) Histograms show the corresponding discrimination index (mean ± SD) for the data shown in A. *p < 0.01. As a control, behavioral analyses were performed on mice without injection.

C57BL/6 male mice received stereotaxic subcortical injections of either oligomeric, fibrillar, or monomeric tau in the right hemisphere and PBS buffer in the left hemisphere, a scheme summarized in Figure 1A, and then we tested the animals in the novel-object recognition task. To compare our methods with the standard heparin method, an additional control group was injected with tau fibrils prepared according the standard heparin protocol [30,31]. Figure 1B shows the precise site of the injections, just above the CA1 region of the hippocampus. We chose this site for the injection according to previous studies that described large number of NFTs and extensive neuron loss from the CA1 region of the hippocampus in patients with AD in comparison with age-matched controls [32-35]. Tau oligomers and fibrils were characterized by AFM (Figure 1D, E and 1F), and the oligomers were purified by FPLC (Figure 1C) before injection. The oligomeric species molecular weight indicates that it represents a tau dimer/trimer, as previously described [36]. We injected the tau preparations and PBS into the C57BL/6 mice 24 h before training in an arena containing two objects that they could explore freely (familiarization phase). Six hours later, we exposed the mice to one familiar and one new object (test phase). Mice injected with tau oligomers were unable to distinguish the new object, with no significant difference in the percentage of time spent investigating both objects (Figure 2A), and displayed a discrimination index significantly lower than that of the other three groups (Figure 2B). Neither monomeric nor any of the fibrillar tau animals showed memory deficits in this task (Figure 2A and 2B). These data indicate that tau oligomers acutely disrupt anterograde memory storage, which parallels the anterograde memory deficits in early-stage AD patients who are unable to store newly-acquired information [37].

### Tau oligomers induced neuronal degeneration

**Figure 3:**
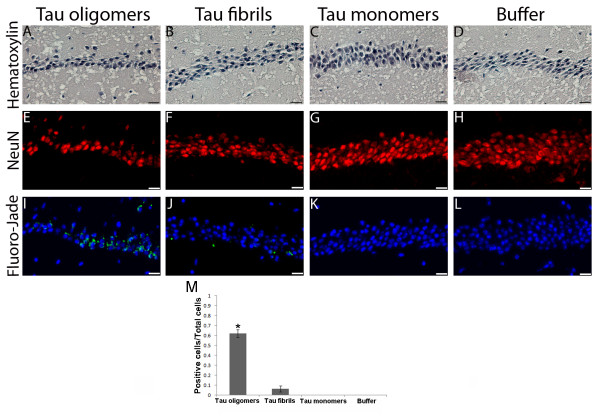
**In vivo neurotoxicity of tau oligomers**. A-D) Nuclear staining (Hematoxylin) of the hippocampal region CA1 from the hemispheres of mice injected with tau preparations or PBS (Scale bar = 20 μm). E-H) Neuronal staining using the antibody NeuN show neuronal damage in the area injected with tau oligomers (E) in comparison with the other hemispheres injected with fibrils (F) monomers (G) and PBS (H) (scale bar = 10 μm). (I-L) Higher toxicity of tau oligomers versus fibrils, monomers and PBS. Neurodegenerative pyramidal cells in the CA1 hippocampal region were stained with Fluoro-Jade B, emitting green fluorescence (scale bar = 10 μm). M) Number of Fluoro-Jade B positive cells/Total number of cells in the CA1 region, 63% of cells showed signs of damage in the group injected with tau oligomers, compared to less than 6.5% in the group injected with tau fibrils. Data are represented as the mean ± SE. *p < 0.01.

Next, we investigated the effects of different forms of tau in neuronal degeneration. We harvested the brains from six animals that underwent behavioral testing and then fixed them in 4% buffered paraformaldehyde, and embedded in paraffin. Cell damage was detected only in the group injected with tau oligomers and only in the CA1 region of the hippocampus in the right hemisphere (Figure 3A, E and 3I. 

To determine cell damage, we performed nuclear staining using Hematoxylin (Figure 3A-D) and neuronal staining was performed using NeuN antibody (Figure 3E-H). In both cases, tau oligomers were highly toxic, producing great destruction of the pyramidal layer of neurons in the hippocampal region, underneath the injection site (Figure 3A and 3E). Tau fibrils also show some degree of toxicity, but much less than tau oligomers (Figure 3B and 3F). We detected cell damage solely in the group injected with tau oligomers and only in the CA1 region of the hippocampus in the right hemisphere (Figure 3). None of the mice displayed any damage in the hippocampus of the left hemisphere (Figure 3D, H, and 3L), demonstrating that the toxicity produced by tau oligomers is located in the area of injection, even at least 30 hours post-injection. The oligomers that diffused to other areas of the hippocampus did not produce neuronal damage (Additional File 1, Figure 1), probably because of the low concentration of oligomers in these areas. To investigate neurodegeneration, we stained sections with Fluoro-Jade B, which is a fluorescein derivative that specifically labels dying neurons in the brain [38,39]. As seen in Figure 3, neurons exposed to tau oligomers exhibit intense Fluoro-Jade B labeling, which indicates a high level of neurodegeneration. It was also possible to see some labeling in the neurons treated with fibrils (Figure 3), but it is very low in comparison with the group treated with oligomeric tau. Fluoro-Jade B positive cells were counted and nearly 60% of the neurons in the CA1 region, showed signs of damage in the group injected with tau oligomers, compared to less than 7% in the group injected with tau fibrils. Fluoro-Jade B binds to polyaminergic products of cellular degeneration, such as spermidine, cadaverine, and putrescine [38,39]. These data demonstrate that tau oligomers produce neuronal damage of the CA1 region of the hippocampus, which is also typical in patients with AD [34,35]. Using these same parameters, we found that tau fibrils present some neuronal damage in vivo, but clearly lower damage than tau oligomers (Figure 3). The fact that tau fibrils present some toxicity may be because the fibrils were partially broken-down into smaller, oligomeric aggregates. In addition, we must take into consideration that the fibrils were directly prepared from tau oligomers, so there remains a possibility that the injected solution contained a small amount of tau oligomers and/or prefibrillar oligomers that could explain the detectable amount of toxicity. To elucidate this point, as we mentioned in the previous section, we injected into another group of mice tau fibrils (0.9 mg/mL) prepared using the heparin protocol [30,31]. This is a well-described procedure to produce highly pure tau fibrils. Mice did not present any memory impairment (Figure 2) or Fluoro-Jade B positive staining (data not shown); suggesting that the mild cellular damage observed in the group injected with fibrils prepared from oligomers was due to the presence of oligomers in the preparation. Using the fibrils prepared with heparin showed no toxicity even when we injected twice the amount of tau fibrils by using the same volume at twice the original concentration of each tau species (1.8 mg/mL). As observed in the original experiments, only the mice injected with tau oligomers exhibited memory impairment (Additional File 1, Figure 2). These data suggest that the amount of oligomers in the tau fibrils is minimal; therefore, it has no considerable toxic effects and does not cause memory deficits in the mice.

### Loss of synaptic markers in mice injected with tau oligomers

Early changes in AD brains include loss of synapses. This precedes the anomalous aggregation of protein (amyloid plaques, NFTs) and correlates with cognitive dysfunction [40,41]. The role of tau in this context has yet to be ascertained, but several studies using in vivo models gave evidence of the relevance of tau in synaptic loss and synaptic dysfunction [11]. In the P301S mutant human tau transgenic mice, Yoshiyama et al. detected loss of hippocampal synapses and impaired synaptic function [15]. A relevant point of that study is that synaptic dysfunction appears before the formation of NFTs, which gives evidence implicating synaptic pathology as an early neurotoxic consequence of pathogenic human tau expression culminating in progressive neurodegeneration [15]. In a recent study, Polydoro et al. showed through behavioral and electrophysiological experiments for the first time that an accumulation and aggregation of non-mutant tau isoforms might cause cognitive and synaptic dysfunction [14].

**Figure 4:**
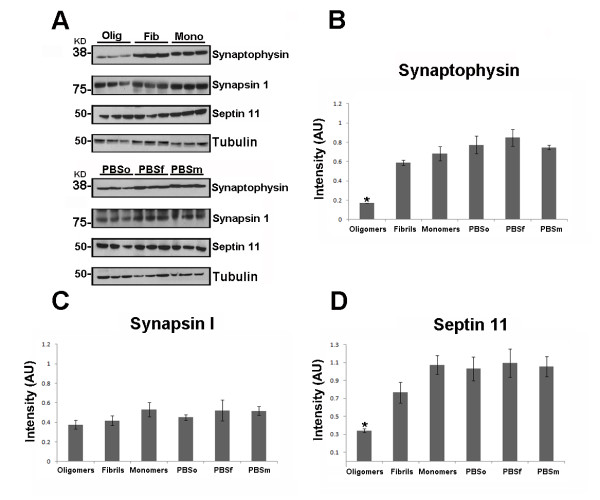
**Tau oligomers induce synaptic dysfunction**. (A) Representative Western blot of mouse hippocampus homogenate. The levels of synaptophysin, synapsin-1, and septin-11 were measured by band quantification and normalized with the levels of tubulin. PBSo indicates representative bands of hippocampal area injected with PBS in mice also injected with tau oligomers, PBSf indicates PBS injection in mice also injected with tau fibrils, and PBSm indicates PBS injection in mice also injected with tau monomers. (B) Synaptophysin levels were significantly lower in the hemispheres injected with tau oligomers in comparison with the ones injected with fibrils, monomers, or PBS. (C) No significant differences in the levels of synapsin-1 were observed. (D) Only the hemisphere injected with tau oligomers presents a decrease in the level of septin-11. Data are represented as the mean ± SE. *p < 0.01, n = 6

**Figure 5:**
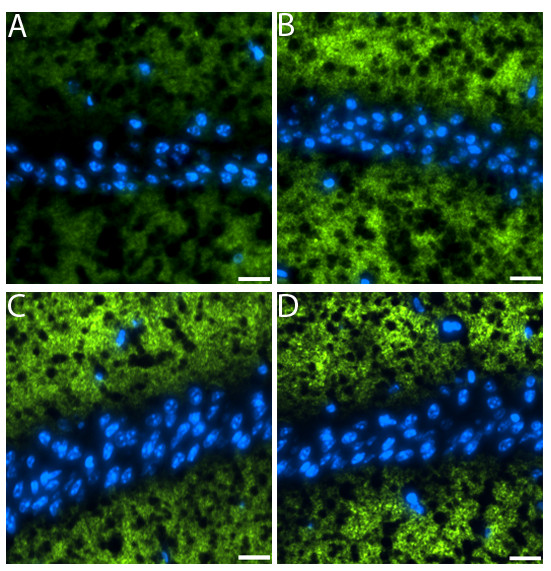
**Decrease in synaptophysin in mice injected with tau oligomers**. Brain sections were stained with antibody to the presynaptic marker synaptophysin (green) nuclear staining with DAPI (blue). All images were taken from the CA1 region. Hemispheres injected with tau oligomers exhibited a decreased in synaptophysin (A). No decrease in the signal was observed in the hemispheres injected with fibrils (B), monomers (C), and PBS (D). (Scale bars = 10 μm.)

To assess the effect of different tau species on synapses using biochemical and histochemical analyses, we measured the levels of several proteins related to synaptic function. These proteins included the synaptic vesicle-associated proteins, synaptophysin, synapsin-1, and septin-11. Synaptophysin is a vesicle-bound presynaptic protein, synapsin-1 regulates the reserve pool of synaptic vesicles [42], and septin-11 is involved in vesicle trafficking and may play a role in synaptic connectivity. For biochemical analysis, Western blot assays were performed using the PBS-soluble brain fraction (six mice per group) and subsequently the relative amount of protein was quantified by densitometry and normalized with tubulin. We observed a clear decrease in synaptophysin in the group injected with tau oligomers in comparison with the other two groups (Figure 4B). We then confirmed this result by performing immunohistochemistry, where we saw a decrease of synaptophysin density in the CA1 region of the brain hemisphere injected with oligomers (Figure 5). No changes in synaptophysin level occurred in the brains injected with fibrils or monomers, or in any hemisphere injected with PBS. Thus, pre-synapses seem to be affected by tau oligomers in the CA1 region of the hippocampus. Similar to the synaptophysin measurements, the levels of septin-11 were significantly lower in the hemisphere injected with tau oligomers in comparison with the hemispheres injected with fibrils or monomers (Figure 4D). This result indicates that tau oligomers may affect trafficking and microtubule stability. In the case of synapsin-1, we observed no statistical differences in the levels of this protein between the different groups (Figure 4B). This result suggests that tau aggregates do not affect the maintenance of the reserve pool of synaptic vesicle. It is important to mention that in all measurements, with the three different synaptic markers, we did not observe any differences between any of the hemispheres injected with PBS and the hemispheres injected with tau monomers (Figure 4).

### Evidence of mitochondrial dysfunction in mice treated with tau oligomers

In AD, mitochondrial abnormalities occur early in the pathogenic process and likely play a significant role in disease progression. Several studies have demonstrated markedly reduced levels of mitochondrial proteins and activity [43-45] in the brains of AD patients. Recently, using transgenic mice overexpressing the P301L mutant human tau protein, David et al. demonstrated mitochondrial dysfunction by proteomic and functional analysis [46]. Specifically, they demonstrated reduced NADH-ubiquinone oxidoreductase (complex I) activity and impairment of mitochondrial respiration, and ATP synthesis (complex V) with age in P301L mice. Quintanilla et al. demonstrated that inducible expression of tau truncated at Asp-421, to mimic caspase cleavage, induced mitochondrial fragmentation and loss of mitochondrial membrane integrity [47]. In this study, we examined the relationship between possible pathological forms of tau and mitochondrial dysfunction.

**Figure 6:**
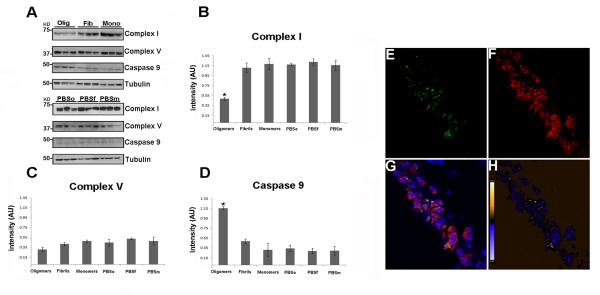
**Tau oligomers induce mitochondrial alterations**. (A) Representative western blot of mouse brain homogenate. The levels of complex I, complex V, and caspase-9 were measured by band quantification and normalized with the levels of tubulin. Abbreviations are as in Fig. 4. (B) Complex I levels were significantly lower in the hemisphere injected with tau oligomers in comparison with the ones injected with fibrils or monomers. (C) No differences in the levels of complex V were observed in any of the groups. (D) Caspase-9 activation was significantly higher in tau oligomer groups over both tau fibril- and monomer-injected groups. (E-G) Double staining between human tau-specific antibody HT7 (green fluorescence) and mitochondrial porin antibody (red fluorescence) demonstrates the internalization of tau oligomers in CA1 cells and their interaction with the mitochondria. (H) The co-localization of tau oligomers with mitochondria was confirmed by ICA of the signal. Data are represented as mean ± SE. *p < 0.01, n = 6.

Studies that correlate the stage of aggregation of tau with mitochondrial abnormalities are in short supply. For this reason, we measured the levels of mitochondrial complexes I and V in all groups. We saw decreased levels of NADH-ubiquinone oxidoreductase (electron transport chain complex I) in the hemispheres injected with tau oligomers in comparison with the ones injected with fibrils or monomers (Figure 6B). This result correlates with previous studies of AD brains, suggesting that modifications of mitochondrial encoded complex I subunit mRNA [48,49], reduction in protein levels of the 24- and 75-kDa subunits of complex I [45], and other mitochondrial dysfunction effects [46] can arise owing to tau accumulation in the absence of massive formation of NFTs. In the case of complex V levels, we did not observe any statistical difference between any of the groups (Figure 6C), demonstrating that tau oligomers do not acutely affect the initial state of ATP synthesis. Our results suggest that tau oligomers initially affect complex I activity and complex V could also be affected in a later state directly or indirectly by the action of tau oligomers.

The fate of injected tau oligomers was investigated (Figure 6E-H), double staining using the mouse monoclonal antibody HT7 (Figure 6E), which recognizes human tau but not murine tau [50], and anti-porin antibody, as a marker for mitochondria (Figure 6F); co-localization between tau oligomers and mitochondria in CA1 cells (Figure 6G);. This was confirmed by ICA of the signal (Figure 6H), as described in [51], suggesting a direct interaction between tau oligomers and mitochondria.

**Figure 7:**
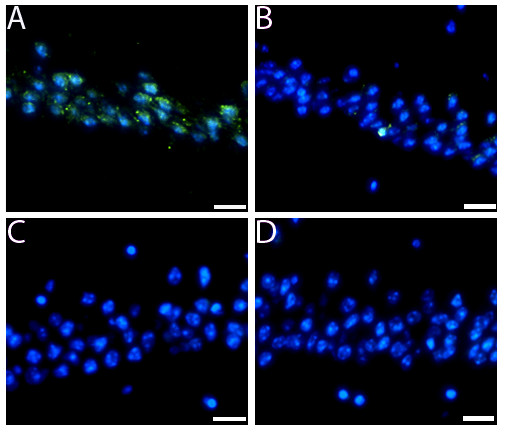
**Caspase-9 activation in hemisphere injected with tau oligomers**. Immunofluorescence using an antibody for pro-caspase-9 was performed. (A and B) Caspase-9 activation (green fluorescence) was observed in hemispheres injected with tau oligomers (A) or fibrils (B). However, the levels of activation were significantly higher in the hemispheres injected with oligomers compared with fibrils, which is not significant. (C and D). No activation of caspase-9 was observed in the hemispheres injected with tau monomers (C) or PBS (D). Nucleus was stained with DAPI (blue). (Scale bars = 10 μm.).

Neuronal commitment to apoptosis may occur through a mitochondrial pathway employing caspase-9 or through an alternative, receptor-mediated pathway involving caspase-8 [52]. Considering the role of mitochondrial dysfunction in AD, we examined the possible activation of caspase-9 by using an antibody that recognizes the active fragment of caspase-9. Western blot analysis demonstrated a considerable activation of caspase-9 in the hemispheres injected with tau oligomers (Figure 6D). By using immunohistochemistry techniques, we confirmed that the activation of caspase-9 is mainly in the hemispheres injected with tau oligomers and in varying levels in the hemispheres injected with fibrils, which was not statistically significant according to the biochemical analysis. We did not observe caspase-9 activation in the hemispheres injected with the monomer or PBS (Figure 7). These results are supported by a recent study demonstrating a closer association between the formation of NFTs than Aβ deposits in the activation of caspase-9. Moreover, the same study concludes that the activation of caspase-9 precedes tangle formation [53]. We also did not detect caspase-8 activation in our study (data not shown).

## Discussion

The role of soluble oligomeric forms of disease-associated protein has received considerable attention in several neurodegenerative diseases because of their association with toxicity [54-56]. There is a growing belief that intermediates in the formation of NFTs encompass the pathogenic forms of tau [11,16,20,57-59]. Herein, we demonstrated that tau oligomers injected in proximity of the hippocampus were responsible for immediate memory impairment in mice by acutely disrupting anterograde memory storage. These observations correlate with previous studies in humans and primates that have shown hippocampal lesions to result in impaired object recognition [60,61]. The neuronal damage produced by tau oligomers in the CA1 region is consistent with previous work, which shows that neurodegeneration in CA1 region occurs prior to the formation of NFTs and the clinical diagnosis of dementia in AD patients [62]. This suggests that tau oligomers could be responsible for neuronal death in the CA1 region in AD or other tauopathies. In the specific case of AD, NFTs progressively spread throughout the brain in an anatomically stereotypical manner [63,64]. Based on these and several other studies, it has been postulated that tau proteins spread in a prion-like mechanism in the tauopathies [65-67]. Clavaguera et al.'s observations support this concept by demonstrating that intracerebral injections of brain extract from mice with a filamentous tau pathology (P301S mutation) induces the formation and spreading of tau aggregates. The pathology spread from the injection site to neighboring brain regions 15 months post-injection in transgenic mice for human wild-type tau (ALZ17 mouse model) [66] and 12 months post-injection in wild-type mice.

The neurodegeneration produced by tau oligomers occurred exclusively in the regions at the injection site (CA1 region). In future studies it will be necessary to establish several time points and work with transgenic mouse models that express tau in order to determine if tau oligomers induce endogenous tau aggregation and spread into further areas of the brain. Nevertheless, our results are in accordance with previous in vitro studies that reveal extracellular tau to be toxic to neuronal cells, and aggregated tau to be capable of propagation from the outside to the inside of a cell [68-70]. On the basis of their results, Gomez-Ramos et al. suggested that NFTs, or other compounds released by degenerating neurons, may accumulate in the extracellular space and this could be toxic to the surrounding cells [68]. Frost et al. showed that cultured cells take up extracellular aggregated tau, but not monomers, and that the internalized tau aggregates induce fibrillization of intracellular tau. Moreover, they observed that newly aggregated intracellular tau transfers between co-cultured cells [69]. If we take into consideration these studies and the novel data presented in our study, we can postulate that tau oligomers generated intracellularly could be released either by binding and local rupture of the membrane, or after cell death. The oligomers in the extracellular space could be taken-up by healthy neurons in the vicinity disrupt normal activity and stimulate further aggregation of functional monomeric tau. Our results show that tau oligomers are co-localized with mitochondria and display intraneuronal punctuate staining with HT7 antibody, indicative of the internalization of injected tau oligomers.

Others in the field propose that a connection between tau pathology and mitochondrial impairment is prevalent [46,47]. Several studies suggest that in AD, mitochondrial abnormalities occur early in the pathogenic process and likely play a significant role in disease progression. In our study, we observed that the injected oligomers co-localized with the mitochondrial marker porin, providing further evidence of a pathological relation between oligomeric tau and mitochondrial dysfunction. Tau oligomers may impair microtubule stability and trafficking, which can affect the distribution of various organelles, including mitochondria. Mitochondria travel for long distances to fulfill the high-energy demand of synapses; thus, inhibition of transport disrupts the supply lines for energy. An alternative approach of how tau can affect synapses could be the direct inhibition of energy production through the mitochondria in the synapses. We found decreased levels of complex I in the hemispheres injected with tau oligomers as compared with the brains injected with fibrils or monomers. This decrease in complex I may lead to severe energy impairments in synapses, as a reduction in the activity of complex I of only 25% impairs energy metabolism in synaptic mitochondria [71], whereas a reduction of complex I activity by 72% is necessary to impair energy metabolism in non-synaptic mitochondria [72]. We did not observe statistical significances in complex V levels between any of the groups. This relates well with the data presented by David et al., where age affects complex V, but decreases in complex I occur early in the disease process [46], suggesting tau oligomers could affect complex V in a later stage of pathology, either directly or indirectly. Overall, according to our results, we can postulate that tau oligomers inhibit energy production through complex I, which can produce synaptic alterations in synaptic-localized mitochondria.

In AD brains loss of synapses precedes NFTs formation and correlates with cognitive dysfunction [40,41]. Even though the role of tau in this context remains unclear, in studies of other models of tauopathies and in several tau mouse models [14,15], the authors reported synaptic damage [73-75]. A recent study showed that missorting of tau in neurons causes degeneration of synapses, and the authors suggest that synaptic decay could be caused by some oligomeric form of tau that is not currently well defined [76]. Moreover, a very recent study by Kimura et al. demonstrated that in wild-type tau Tg mice, an early stage of tau aggregation (sarkosyl-soluble) produced synaptic loss and memory impairment [77]. To further investigate synaptic alternations, we measured levels of characterized synaptic markers: synaptophysin and septin-11, which are involved in synaptic vesicle docking and trafficking, and synapsin-1, which is associated with the vesicle reserve pool. We found that only tau oligomers caused a decrease in synaptophysin and septin-11; however, no difference existed in the levels of synapsin-1 between all the groups. These results suggest that tau oligomers can affect presynaptic density and neuronal trafficking, but do not interfere with the reserved pool of synaptic vesicles. Another effect of mitochondrial dysfunction is the activation of the mitochondrial apoptotic pathway, measured by activation of caspase-9. We measured an increase in caspase-9 activation in the hemispheres injected with tau oligomers, but not in the PBS-injected hemispheres. Earlier studies demonstrated the presence of activated caspase-9 in synaptosomal preparation of AD brains [78]. A possible explanation for the caspase activation could be that tau oligomers accumulate at the mitochondrial membrane, resulting in the release of cytochrome C, which leads to caspase-9 activation through a complex with apoptotic peptidase activating factor-1 (Apaf-1) [79]. This mechanism has been suggested for other amyloidogenic proteins such as α-synuclein, Aβ, and PrP [80,81]. Even more, recently de Calignon et al, demonstrated that caspase activation precedes NFTs formation, suggesting that soluble tau species, rather than fibrillar tau, may be the critical toxic moiety underlying neurodegeneration [82].

The field of tau oligomers and other pre-filament tau aggregates is becoming an extremely important area of research in neurodegenerative diseases. Thus, understanding the negative impact of tau oligomers in neuronal damage, specifically in reference to important cellular mechanisms, such as mitochondrial and synaptic function, will likely be of great importance to understand the relevant disease processes and progression in AD and other tauopathies. These results support the hypothesis that tau oligomers are the acutely toxic species in tau aggregation, and the disruption of mitochondrial membrane, the activation of the apoptotic-related caspase-9, and reduction in complex I levels can cause this toxicity, leading to the inhibition of synaptic energy production due to tau oligomers. These cellular dysfunctions may underlie the synaptic dysfunction and memory impairments observed with tau oligomers in this study.

## Competing interests

RK is the founder of ConImm, Inc. and has patent applications on the compositions, methods and reagents related to tau oligomers.

## Authors' contributions

RK and CL designed the experiments. CL, DC, AC and US performed the experiments. RK, CL prepared the figures and analyzed the data. CL, GJ and RK wrote the paper. All authors read and approved the final manuscript.
